# Sakuranetin, A Laxative Component from Peach Leaves and Its Intervention in Metabolism

**DOI:** 10.3390/ijms26178112

**Published:** 2025-08-22

**Authors:** Ping Wang, Yi Song, Haixin Jiang, Chenyuan Qi, Xubo Zhang, Disheng Wang, Luqi Li, Qiang Zhang

**Affiliations:** 1 Shaanxi Key Laboratory of Natural Products & Chemical Biology, College of Chemistry & Pharmacy, Northwest A&F University, Yangling 712100, China; 180406wp@nwafu.edu.cn (P.W.); yisong@nwafu.edu.cn (Y.S.); 2544945256@nwafu.edu.cn (H.J.); qichenyuan@nwafu.edu.cn (C.Q.); zhangxubo@nwafu.edu.cn (X.Z.); 2020011944@nwafu.edu.cn (D.W.); 2 Life Science Research Core Services, Northwest A&F University, Yangling 712100, China; liluqi167@nwafu.edu.cn

**Keywords:** *Prunus persica*, Sakuranetin, gut health, transcriptome, target analysis

## Abstract

Peach (*Prunus persica*) leaves, usually discarded in traditional Chinese medicine, were explored as a source of laxative agents. Using zebrafish larvae for bioactivity-guided fractionation, we isolated a single active flavanone that was identified by NMR and HR-MS as Sakuranetin. In vivo assays demonstrated that Sakuranetin (10–25 µM) accelerated intestinal transit in a dose-dependent fashion; at 25 µM, 64.8% of the fluorescent intestinal content was expelled. Untargeted LC-MS metabolomic analysis revealed significant perturbations in serine biosynthesis and N-glycan precursor biosynthesis, suggesting energetic rewiring of enterocytes. RNA-Seq analysis highlighted *gnat1* as the most responsive gene, and molecular docking predicted a stable Sakuranetin–Gnat1 complex with a binding free energy of −8.7 kcal/mol. Concurrent down-regulation of *rho* transcripts indicated suppression of inflammatory signaling that often accompanies constipation. Our findings identified Sakuranetin as a potent promoter of gut motility and position the otherwise wasted peach leaves as an untapped botanical resource for developing anti-constipation therapeutics.

## 1. Introduction

Laxatives are used primarily to manage functional constipation [1], to prevent and treat opioid-induced constipation (OIC) [2], to facilitate perioperative recovery of bowel function [3], and for tailored use in frail or institutionalized older adults [4]. Current pharmacological management of constipation relies chiefly on osmotic and stimulant laxatives. Osmotic agents such as polyethylene glycol establish a hypertonic intraluminal milieu that draws water into the bowel, softens stool, and accelerates evacuation. Stimulant preparations including bisacodyl and the anthraquinone glycosides in Senna act directly on the enteric nervous system to boost peristalsis and fluid secretion. While generally effective, chronic or high-dose use (particularly of stimulants) can provoke tolerance, physiological dependence, and, upon abrupt withdrawal, peripheral edema [5]. In recognition of these drawbacks, newer mechanism-based drugs are being developed. Secretagogues like lubiprostone activate chloride channels on intestinal epithelial cells, promoting isotonic fluid release without directly stimulating smooth muscle [6]. Ileal bile acid transporter inhibitors (e.g., elobixibat) enhance colonic secretion by allowing bile acid accumulation [7]. Nevertheless, the quest continues for agents that combine robust pro-motility activity with improved safety and reduced risk of dependence.

Natural products derived from traditional Chinese herbs have gained increasing attention as safe and effective alternatives to conventional laxatives, which often cause adverse effects such as electrolyte imbalances or dependency [8,9]. Cathartic herbs, such as *Rheum palmatum* (rhubarb), *Astragalus membranaceus* (Huangqi), and *Cistanche deserticola* (Roucongrong), have demonstrated multifaceted therapeutic potential by synergistically modulating gut microbiota composition, enhancing intestinal motility, and alleviating oxidative stress and inflammation [8]. Emerging evidence further highlights their roles in sustaining gastrointestinal health through bioactive constituents—such as anthraquinones, dietary fibers, and phenolic acids—that interact with intestinal glucose absorption pathways and microbial metabolites [10,11,12]. Sennoside A and Sennoside B, primarily extracted from *Rheum* and *Senna*, are well-known stimulant laxatives. They function by promoting intestinal peristalsis and increasing intestinal secretion. However, prolonged use may lead to tolerance and adverse effects [13,14]. Emodin, an anthraquinone compound found in *Rheum* and *Cassia*, exerts a laxative effect by inhibiting intestinal water absorption [15]. Pterostilbene, a natural polyphenol present in blueberries, has demonstrated laxative effects in slow-transit constipation mouse models, potentially through gut microbiota regulation [16]. Multiflorin A, an active component of *Prunus semen*, increases intestinal osmotic pressure by inhibiting glucose absorption, leading to bowel movements [10]. Diosmetin, found in certain plant extracts, induces laxation at low doses through cholinergic and histaminergic pathways-mediated intestinal spasmodic contractions [17].

**Figure 1:**
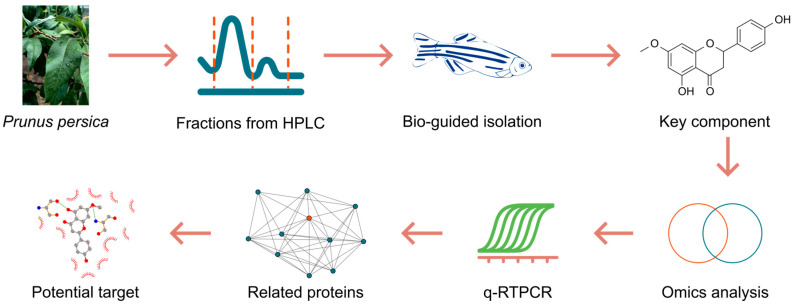
Workflow of the present study.

*Prunus persica* (L.) Batsch, commonly known as the peach tree, has a long history of medicinal use in East Asia. In the Chinese Pharmacopoeia, the dried seeds (*Persicae Semen*, “Tao Ren”) and the young twigs (*Persicae Ramulus*, “Tao Zhi”) are the officially listed medicinal parts, traditionally prescribed for blood stasis-related disorders and inflammatory conditions. The leaves remain as a non-medicinal by-product and are largely discarded during harvesting and processing. Given the plant biosynthetic capacity and the growing emphasis on sustainable use of herbal resources, the leaves represent an under-explored reservoir of bioactive constituents that could be valorized for novel therapeutic applications. Accordingly, we employed small animal (zebrafish larvae) models and bioactivity-guided isolation to mine a key laxative constituent from peach leaves, evaluated its modulation of in vivo metabolism, and identified putative molecular targets by integrating transcriptomic profiling with molecular docking (Figure 1).

## 2. Results

### 2.1. Bio-Guided Isolation of Laxative Component in P. persica Leaves

**Figure 2:**
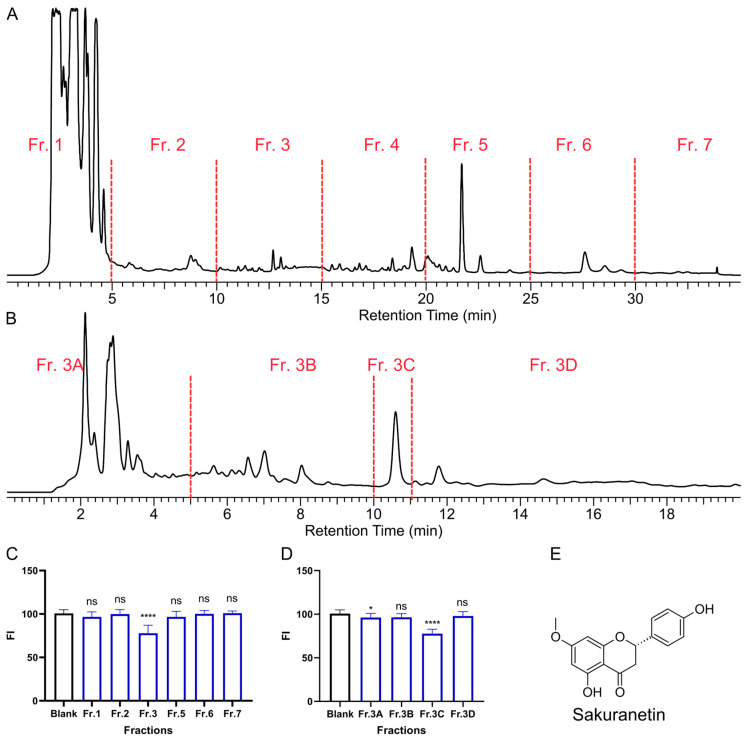
Bioactivity-guided isolation of laxative components from the leaves of *P. persica*. HPLC profile and separation of the extract of peach leaves (**A**) and Fr. 3 (**B**). Laxative effects of 20 μg/mL extract fractions (**C**) and 20 μg/mL separated fractions (**D**); *n* = 10; **** *p* < 0.0001, * *p* < 0.05; ns, not significant; compared to the blank control. (**E**) Chemical structure of Sakuranetin.

During our screening of TCMs for agents that promote intestinal peristalsis, we found that *P. persica* leaves (Figure S1 in the Supplementary Materials) demonstrated notable activity in this regard. To rapidly isolate the key bioactive constituents, the crude extract was subjected to HPLC separation (Figure 2A,B), yielding seven fractions designated as Fr. 1~Fr. 7 (Figure 2A). The laxative effect can be determined by the residual fluorescence intensity (FI) in zebrafish larvae stained with Nile Red solid (Figure 2C). Nile Red is a non-absorbable fluorescent tracer that stays within the intestinal lumen and is excreted, so its signal reliably tracks transit [18]. In transparent zebrafish larvae, gut fluorescence is imaged noninvasively. Among these, Fr. 3 exhibited significant stimulatory activity on intestinal peristalsis. Subsequent bioactivity-guided fractionation (Figure 2B,D) led to Fr. 3C as the principal active fraction (20 μg/mL). This fraction exhibited a single chromatographic peak; after purification, ^1^H and ^13^C NMR, together with HR-ESI-MS (Figure S2 in the Supplementary Materials), identified the active compound as Sakuranetin (Figure 2E) [19]. The compound had a single chiral center. Its specific rotation was negative, and comparison with literature [20] indicates that it was (−)-(S)-Sakuranetin.

### 2.2. Laxative Activity of Sakuranetin

**Figure 3:**
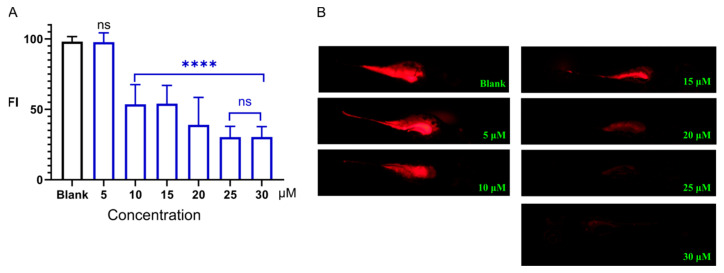
(**A**) Dose–effect relationship of the laxative action of Sakuranetin, *n* = 10. **** *p* < 0.0001; ns, not significant; compared to the control group. FI, fluorescence intensity. (**B**) Images of zebrafish larvae exposed to Sakuranetin, stained with Nile Red.

We further investigated the dose–response relationship of Sakuranetin (Figure 3). At a concentration of 5 μM, Sakuranetin showed no significant difference in FI, compared to the blank control group. However, upon exposure to Sakuranetin at concentrations ranging from 10 to 30 μM, there was a marked decrease in fluorescence intensity (*p* < 0.0001), compared with the blank control group. This indicates that Sakuranetin promoted the discharge of dye, thereby facilitating intestinal peristalsis and exerting a laxative effect. The results also suggested a dose-dependent relationship, with higher concentrations of Sakuranetin leading to more pronounced effects. There was no significant difference in the laxative activity between 25 μM and 30 μM Sakuranetin. Raising the concentration to 30 μM produced no statistically significant additional reduction beyond that observed at 25 μM (*p* > 0.05), presumably because the intestinal fluorescent content was already near its minimum, limiting further differentiation in the fluorescence-based readout. These data provide compelling evidence of Sakuranetin as a potential laxative agent, as observed in the zebrafish larval model.

**Figure 4:**
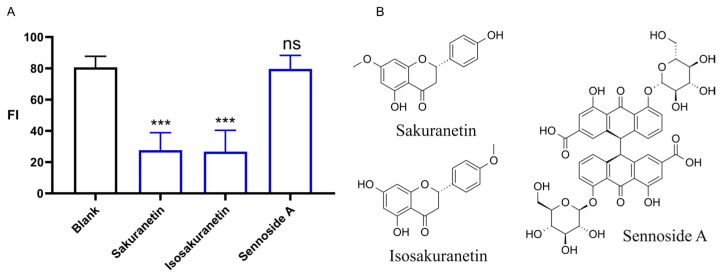
Laxative activities (**A**) and chemical structures (**B**) of Sakuranetin, Isosakuranetin, and Sennoside A; *n* = 10; ***, *p* < 0.01; ns, not significant;.compared to the blank control.

We also conducted a comparative analysis of the effects of three natural products, including Sakuranetin, Isosakuranetin, and Sennoside A, on promoting intestinal motility at a concentration of 20 μM (Figure 4). Both Sakuranetin and Isosakuranetin are structurally similar natural products belonging to the class of dihydroflavonoids. Sennoside A, on the other hand, is a well-known natural laxative agent. The results indicated that both Sakuranetin and Isosakuranetin significantly enhance intestinal propel activity, facilitating the excretion of the Nile Red dye within the body. Conversely, the effect of Sennoside A was not significant in promoting intestinal motility.

Sennoside A is a well-known natural laxative agent that exerts its effects through intervention in the gut microbiota, specifically through the modulation of lactobacillus of the genera of *Lactobacillus*, *Romboutsia*, *Akkermansia*, and UCG_005 [21]. The mechanism also involves the inhibition of water channel proteins, such as AQP3 and AQP7, in the epithelial cells of the colon, which reduces the reabsorption of water in the intestines, thus increasing fecal moisture content [13]. However, this effect is not applicable to larval zebrafish, which have not yet established their gut microbiota and, therefore, are unable to leverage the action of Sennoside A in an aquatic environment to suppress intestinal water reabsorption.

### 2.3. The Effects of Sakuranetin on Lipid Accumulation

**Figure 5:**
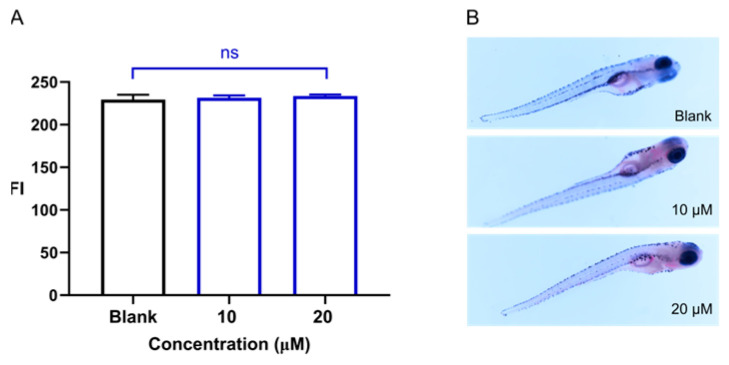
Effect of Sakuranetin on lipid accumulation in vivo, as assessed by Oil Red O staining ((**A**), fluorescence intensity; (**B**), images); ns, not significant;.compared to the blank control. No significant changes were detected after being exposed to Sakuranetin. Moreover, Sakuranetin exhibited no apparent toxicity in zebrafish larvae.

We further examined the impact of Sakuranetin exposure on lipid accumulation in zebrafish larvae. Compared with the control group, treatment with Sakuranetin did not induce significant alterations in lipid deposition, nor did it elicit any observable toxicity (Figure 5).

### 2.4. Intervention of Sakuranetin on Metabolome In Vivo

**Figure 6:**
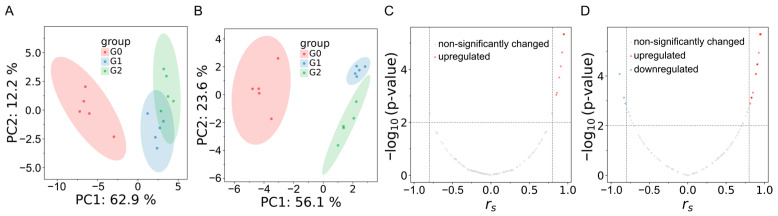
Metabolism analysis. G0, blank control; G1, treated with 10 μM Sakuranetin; G2, treated with 20 μM Sakuranetin. (**A**,**B**) PCA analysis of metabolite expression levels detected on positive (**A**) and negative (**B**) LC-MS/MS. (**B**) PCA analysis of metabolite expression levels detected on negative LC-MS/MS. (**C**,**D**) Metabolites with correlated expression levels to Sakuranetin treatment concentrations, analyzed by Spearman correlation analysis (|*r*_s_| > 0.8, *p* < 0.01), were detected in positive (**C**) and negative (**D**) modes of LC-MSMS.

To assess whether the laxative effect exerts an influence on systemic metabolism, we collected metabolic profiles from zebrafish following Sakuranetin intervention. We performed untargeted LC-MS metabolomic analysis using high-resolution LC-Orbitrap-MS/MS. In positive- and negative-ion modes, 123 and 110 metabolites were identified, respectively (Tables S1 and S2 in the Supplementary Material). PCA analysis (Figure 6A,B) revealed distinct metabolic changes between the control group (G0) and Sakuranetin-treated groups (G1 and G2, treated with 10 and 20 μM, respectively). The G2 group exhibited greater overall metabolic variation compared to G0, indicating a dose-dependent metabolic shift.

To identify endogenous metabolites exhibiting concentration-dependent responses to Sakuranetin exposure, Spearman monotonicity analysis was performed. This approach enabled the detection of differential metabolites whose expression levels consistently increased or decreased with rising exposure concentrations. As shown in Figure 6C,D, metabolites with significant expression changes correlated with Sakuranetin concentrations. In positive ion mode, 8 metabolites were detected upregulated, while in negative ion mode, 17 metabolites were detected upregulated, and 3 metabolites downregulated, totaling 28 differentially expressed metabolites (Table S3 in the Supplementary Material). These metabolites showed strong positive or negative correlations with Sakuranetin concentrations.

**Figure 7:**
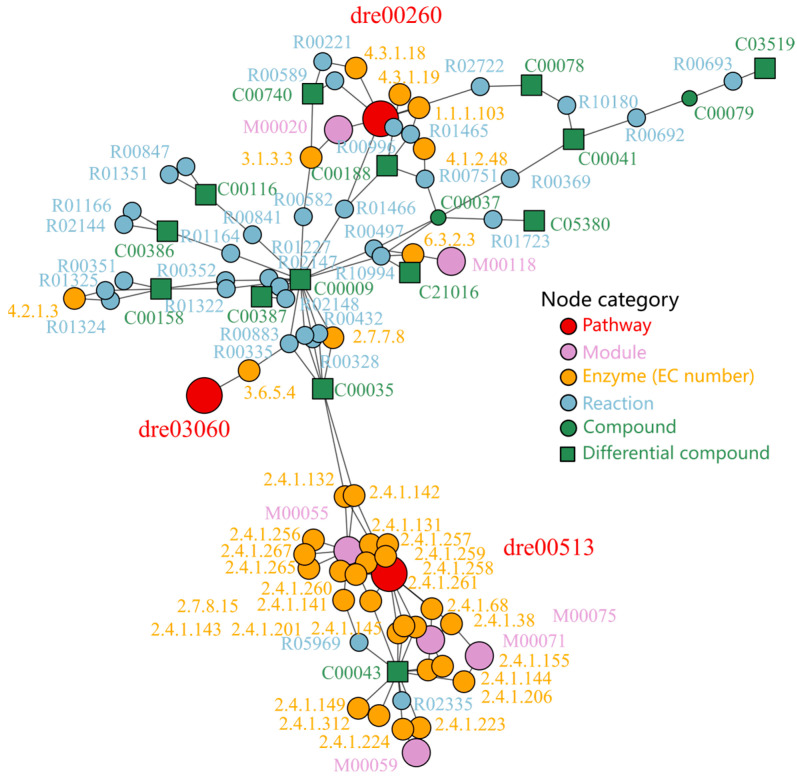
Metabolic network diagram based on FELLA enrichment, *p* < 0.01. The labels are presented as KEGG IDs, and detailed information can be found at https://www.kegg.jp/ (accessed on 23 May 2025).

We then employed the diffusion-based enrichment algorithm implemented in the FELLA package to investigate metabolic alterations in zebrafish larvae based on a set of 28 differentially expressed metabolites identified from the metabolomics profiles. As shown in Figure 7, the analysis revealed that the three most significant pathways were ‘Glycine, Serine, and Threonine Metabolism’ (KEGG ID: dre00260), ‘Various Types of N-glycan Biosynthesis’ (dre00513), and ‘Protein Export’ (dre03060). Based on the network topology, the modules ‘Serine Biosynthesis’ (M00020) and ‘N-glycan Precursor Biosynthesis’ (M00055) were found to be associated with a greater number of differentially expressed metabolites, indicating they are the most significantly affected metabolic modules. These findings underscore the intricate metabolic reprogramming occurring in zebrafish, highlighting the potential impact of these metabolic changes on developmental and physiological processes.

### 2.5. Transcriptomic Profiling Under Sakuranetin Intervention

**Figure 8:**
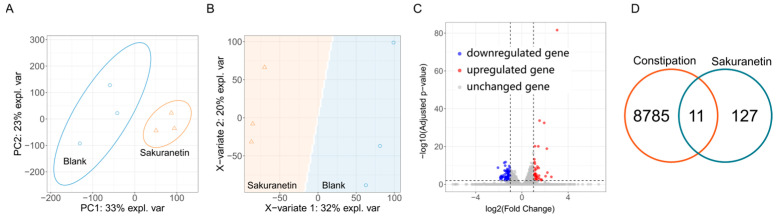
Transcriptomic analysis of zebrafish larvae exposed to 10 μM Sakuranetin and blank control. (**A**) Principal component analysis (PCA) of mRNA expression profiles. (**B**) Partial least squares-discriminant analysis (PLS-DA) of the transcriptomic data. (**C**) Differentially expressed genes (DEGs) identified with |log_2_(fold change)| > 1 and *p* < 0.01. (**D**) Venn diagram illustrating the intersection between DEGs and constipation-associated genes, which were retrieved from the GeneCards database on 26 May 2025.

In the transcriptomic analysis (Figure 8), a total of 25,046 genes were detected. PCA and PLS-DA analyses revealed marked transcriptomic differences between zebrafish with and without Sakuranetin exposure (Figure 8A,B). Of these, 43 genes were found to be upregulated and 95 downregulated, resulting in 138 differentially expressed genes (DEGs) between the Sakuranetin-exposed group and the blank control (Figure 8C). Additionally, 8796 genes associated with constipation were retrieved from the GeneCards (accessed on 26 May 2025) database. Intersection analysis revealed that 11 genes overlapped between the DEGs (Figure 8D and Table S4 in the Supplementary Materials) and the constipation-associated gene set. These 11 genes are therefore implicated in both Sakuranetin treatment and constipation intervention.

**Figure 9:**
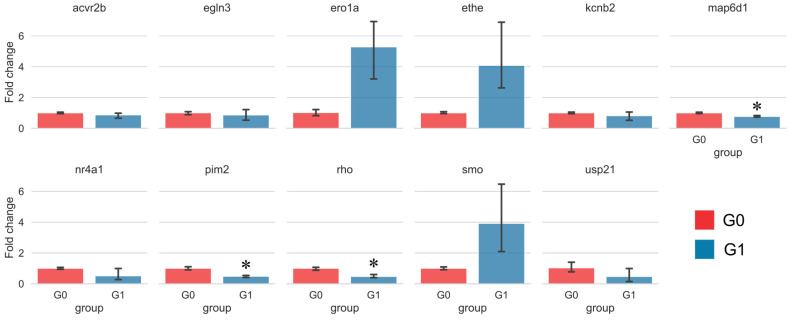
Validation of differentially expressed genes identified by transcriptome analysis using quantitative real-time PCR. * *p* < 0.05, compared to the control group G0.

The expression patterns of the 11 key genes were validated using real-time quantitative PCR (RT-qPCR). Among these, the changes in *map6d1*, *pim2*, and *rho* were statistically significant (Figure 9). Notably, Sakuranetin treatment led to a significant downregulation of these three genes in vivo. This downregulation is associated with enhanced intestinal peristalsis and relief of constipation, suggesting that these genes may serve as related molecular targets of Sakuranetin.

### 2.6. Target Analysis Based on Differentially Expressed Genes

**Figure 10:**
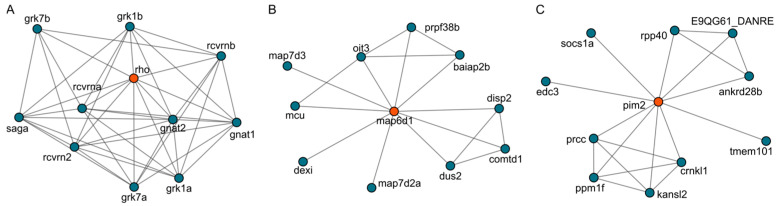
Protein interaction network related to rho (**A**), map6d1 (**B**), and pim2 (**C**), as identified by STRING database analysis.

**Figure 11:**
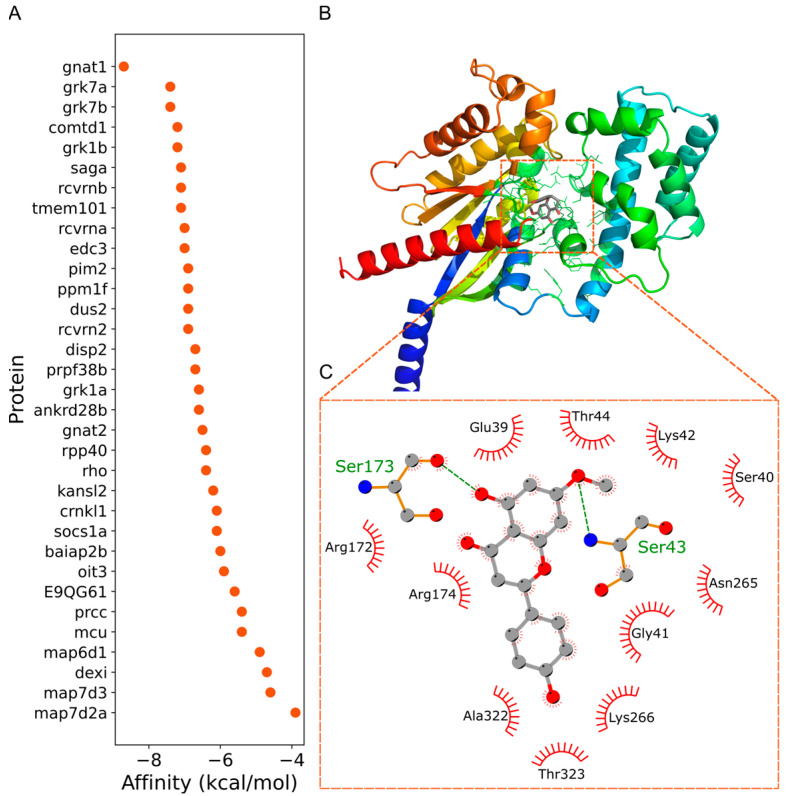
Molecular docking analysis of Sakuranetin with potential targets using PocketVina. (**A**) Binding affinities predicted by PocketVina. (**B**) Predicted binding pose of Sakuranetin with the target protein gnat1. (**C**) Visualization of detailed molecular interactions between Sakuranetin and residues within the gnat1 binding pocket.

To identify potential direct molecular targets, we used the three differentially expressed genes (*map6d1*, *pim2*, and *rho*) as clues and queried the STRING database, retrieving a total of 33 associated proteins and their interactions (Figure 10). The three-dimensional structures of these proteins were subsequently obtained from the AlphaFold database [22]. After performing molecular docking using PocketVina [23], the candidate proteins were ranked according to their binding affinities, as shown in Figure 11A. Among the 33 proteins, molecular docking analysis revealed that Sakuranetin exhibited a notably stronger binding affinity with gnat1 (−8.7 kcal/mol), compared to the other proteins (≥−7.4 kcal/mol). This finding suggests that Sakuranetin could bind to gnat1 and subsequently modulate the expression of rho, thereby enhancing intestinal motility. In addition, Sakuranetin is unlikely to exert its effect on intestinal motility via a single target, as it also displayed relatively strong affinities (<−7.0 kcal/mol) for several other proteins, including grk7a, grk7b, grk1b, saga, and rcvrnb, all of which are known to interact with rho. The binding site and binding mode of Sakuranetin with gnat1 are illustrated in Figure 11B and Figure 11C, respectively. Sakuranetin formed hydrogen bonds with two residues of gnat1, Ser173, and Ser43, with a binding energy of −8.7 kcal/mol. These results indicate that Sakuranetin primarily modulates intestinal motility by targeting gnat1 and subsequently influencing the transcriptional activity of rho.

A considerable binding affinity was also observed between Sakuranetin and tmem101 (−7.1 kcal/mol), a protein interacting with pim2. In contrast, Sakuranetin showed weaker affinities (≥−7.0 kcal/mol) toward proteins associated with map6d1. These results indicate that Sakuranetin can also act, to some extent, on the tmem101 protein, leading to downregulation of *pim2* gene expression.

## 3. Discussion

### 3.1. Sakuranetin Was a Newly Recognized Compound with Laxative Activity

Natural products have long served as a prolific source of bioactive compounds with significant laxative effects. To trace the laxative active components obtained from peach leaves, we used zebrafish larvae as an in vivo activity-tracing model [18]. Zebrafish transit assay offers whole-animal physiological readouts with optical access, enabling non-invasive, quantitative measurement of motility. It is rapid and plate-compatible, requiring modest compound amounts, and exhibits strong pharmacological validation by detecting approved human prokinetics while ignoring inert negatives. The model is cost-efficient and consistent with 3Rs, making it ideal for early screening prior to rodent confirmation.

Our findings identify a new dihydroflavone-type laxative compound that is distinct from prodrugs such as sennoside A, as it does not depend on microbial transformation to exert its activity, even in larval zebrafish with underdeveloped intestinal microbiota. Sennoside A requires bacterial deglycosylation/reduction into rhein anthrone, explaining its lack of efficacy in larval zebrafish with immature microbiota [2,13,14,21]. In contrast, Sakuranetin acted without microbial activation, consistent with the robust activity we observed in larvae. This distinction supports Sakuranetin as a direct-acting motility modulator and highlights potential advantages for conditions with dysbiosis or antibiotic exposure.

Sakuranetin also differs substantially in its mechanism of action from other natural products. Multiflorin A induces osmotic changes by inhibiting glucose absorption [10]. Pterostilbene may act via modulation of the gut microbiota [16]. Diosmetin engages cholinergic/histaminergic pathways [17]. These mechanisms are distinct from those of Sakuranetin.

### 3.2. Sakuranetin Modulated Metabolic Pathways Linked to Intestinal Inflammation

Our metabolomics pointing to glycine/serine/threonine metabolism (KEGG ID: dre00260) provide a physiologically coherent link to both pro-motility and anti-inflammatory effects. Glycine and serine fuel one-carbon flux and glutathione biosynthesis, elevating cellular redox capacity, stabilizing tight junctions, and dampening NF-κB–driven inflammatory signaling [24,25,26,27,28]. Threonine is a key substrate for O-glycosylated mucins; sufficient threonine supports goblet-cell MUC2 production and mucus layer thickness, strengthening the barrier and reducing luminal friction and epithelial irritation. Restoration of antioxidant tone together with barrier reinforcement is associated with improved transit in constipation and attenuated colitis phenotypes. Thus, the observed enrichment of glycine/serine/threonine pathways plausibly reflects enhanced glutathione and mucin biosynthesis, aligning with the measured reduction in inflammatory readouts and the normalization of motility and providing a mechanistic bridge between metabolomic signatures and functional outcomes.

In patients with constipation, the levels of amino acids, such as glycine, serine, and threonine, undergo significant alterations. For instance, glycine and serine levels are decreased in the constipation group, while threonine-associated metabolic pathways—including those involved in bile acid metabolism—are also affected [24]. These metabolic changes may influence intestinal motility by affecting the function of the intestinal mucosal barrier.

In the enriched Glycine, Serine, and Threonine Metabolism pathway (dre00260), metabolomics indicated that Sakuranetin upregulated D-serine and L-threonine while downregulating tryptophan. D-serine exhibits marked anti-inflammatory effects in experimental colitis. Mice pretreated with D-serine show reduced colonic inflammation and T-cell infiltration, particularly diminished Th1/Th17 differentiation—an effect not seen with L-serine or controls. Moreover, D-serine suppresses chronic colitis progression, even when administered after induction [29]. Upregulated L-threonine may enhance mucin synthesis to strengthen the intestinal barrier and stimulate motility via SCFA metabolism [30,31]. Reduced tryptophan may lower inhibitory transmitters and increase excitatory mediators, thereby promoting motility [32]. Thus, the Glycine, Serine, and Threonine Metabolism pathway likely mediates the laxative effect of Sakuranetin.

### 3.3. Sakuranetin Inhibited Rho Gene Level Related to Intestinal Inflammation

Metabolite shifts after Sakuranetin reflect downstream consequences of enhanced peristalsis and typically indicate associations rather than direct causes. To identify upstream drivers, we turned to transcriptomic profiling. By integrating differential expression with constipation-associated genes and validating by qPCR, we prioritized three genes (*rho*, *map6d1*, and *pim2*, Figure 10) as key candidates implicated in Sakuranetin’s action. These genes provide mechanistic leads that complement the metabolomic phenotype and will guide targeted functional assays to test their roles in pathways regulating intestinal motility.

The *rho* gene family plays a pivotal role in intestinal inflammation, gut microbiota dysbiosis, and constipation. Rho GTPases play a critical role in maintaining intestinal tissue homeostasis, particularly within the intestinal mucosal epithelial cells. They influence intestinal barrier function and epithelial repair by regulating cytoskeletal reorganization, intercellular junctions, and signal transduction pathways. For example, the RhoA/ROCK signaling pathway is involved in the regulation of tight junction proteins such as claudin-1 and ZO-1. Dysregulation of this pathway may lead to increased intestinal permeability and inflammation [33]. In addition, RhoB is significantly upregulated in the colonic tissues of patients with ulcerative colitis (UC), and it alleviates colitis symptoms by promoting goblet cell differentiation and epithelial regeneration through inhibition of the Wnt signaling pathway and activation of the p38 MAPK pathway [34].

Sakuranetin is also capable of downregulating the expression of the *pim2* gene, which plays an important role in laxative effects and intestinal health. PIM2 promotes glycolysis by phosphorylating metabolic enzymes such as PFKFB4 and PGK1 [35,36], which may indirectly affect intestinal energy metabolism.

### 3.4. GNAT1 as a Putative Target of Sakuranetin’s Laxative Action

The high accuracy of AlphaFold in protein three-dimensional (3D) structure prediction has greatly expanded our understanding of protein structures, especially for proteins with previously unknown 3D conformations, and has provided an essential data foundation for functional studies and molecular docking analyses [37,38]. Therefore, where computational resources permit, large-scale molecular docking can be employed to identify potential protein targets of specific small molecules, such as Sakuranetin. However, to focus our investigation on proteins modulated by small-molecule intervention, we adopted an integrative approach starting from transcriptomic analysis to obtain differentially expressed genes after Sakuranetin treatment. By mapping these genes to associated proteins using bioinformatics databases, we subsequently performed molecular docking to explore potential targets. This strategy can reduce the blind spots inherent in purely large-scale molecular docking and enhance the relevance of identified targets to Skuranetin intervention.

GNAT1 and GNAT2 are both N-terminal acetyltransferases that exhibit significant interaction. As acetyltransferases and components of the phototransduction pathway, the GNAT1–GNAT2 complex may influence the activity of proteins in the Rho pathway via acetylation modifications [39,40]. Our results indicated that Sakuranetin, by targeting GNAT1, led to the downregulation of the rho gene, which may be related to acetylation modifications of rho, as previously reported in the literature.

### 3.5. Hypothesis, Limitations, and Future Directions

Our metabolomic/transcriptomic signatures, qPCR validation, and docking to a GNAT-family Gα subunit suggest a non-irritant mechanism for Sakuranetin/Isosakuranetin. We propose that these flavonoids promote laxation by shifting epithelial ion transport toward net luminal water—potentiating CFTR-mediated chloride secretion and/or reducing NHE3-dependent sodium absorption while simultaneously engaging gut chemosensory–enteroendocrine signaling to trigger 5-HT release and speed peristalsis. Modulation of inflammation-linked pathways appears to normalize cytokine-driven dysmotility without damaging the mucosa. This model accounts for the observed phenotype and distinguishes it from the irritant, mucus-inducing action of anthraquinone glycosides (e.g., sennoside A). It is also consistent with reports that some flavonoids act as epithelial secretagogues.

Sakuranetin is the principal active constituent, but its direct target remains to be further validated experimentally. Transcriptomics and docking nominate GNAT1, yet this evidence is only suggestive. Whether GNAT1 is a bona fide, druggable target requires orthogonal validation. We propose target-engagement assays (affinity-based probes, CETSA/DARTS, SPR) and genetic perturbation of gnat1 (knockdown/knockout or rescue) in zebrafish and mammalian enteric systems. These studies should establish causality, identify upstream receptors, clarify links to Rho-related signaling, and separate on-target from off-target effects. Translational work should define pharmacokinetics/pharmacodynamics, safety, and efficacy in rodent models of chronic constipation, opioid-induced constipation, and postoperative ileus and in human colonic tissue or organoids. Medicinal chemistry optimization and profiling of microbiome–host interactions may further enhance therapeutic potential and refine the mechanism.

## 4. Materials and Methods

### 4.1. Chemicals and Materials

The dimethyl sulfoxide (DMSO), paraformaldehyde, PBS, and propylene glycol were acquired from Solarbio (Solarbio Life Sciences Co. Ltd., Beijing, China). The adult zebrafish were procured from EzeRinka (EzeRinka Biotechnology Co., Ltd., Nanjing, China). AG RNAex Pro RNA reagent, Evo M-MLV kit, and Premix Pro Taq HS qPCR kit were purchased from Accurate (Accurate Biotechnology Co., Ltd., Changsha, China). The primers were obtained from Sangon (Sangon Biotech Co., Ltd., Shanghai, China).

### 4.2. Bio-Guided Isolation of Laxative Component from Peach Leaves

Dried peach leaves (200 g) were ground and macerated in ethanol (2 L), then sonicated at 40 °C for 1 h and filtered. The filtrate was concentrated to form a crude extract (25.6 g), which was dispersed in water and partitioned with ethyl acetate. The ethyl acetate-soluble fraction was subjected to HPLC fractionation (Figure 2B) using a YMC ODS-A C18 column (5 μm, 250 mm × 4.6 mm, YMC Co., Ltd., Kyoto, Japan). The column was eluted with gradient acetonitrile (40% ~100% over 20 min), followed by acetonitrile for 15 min (total 35 min), which afforded seven fractions collected every 5 min (Fr. 1 ~ Fr. 7). The laxative assay identified Fr. 3 as active. Fr. 3 (192 mg) was further separated on the same system: 50% acetonitrile for 12 min, then acetonitrile for 8 min (total 20 min), with collection windows set around well-resolved peaks (see Figure 2B). Bioassay assigned activity to subfraction Fr.3B (27 mg), which, upon further purification, yielded Sakuranetin (21 mg).

Sakuranetin: white amorphous powder; [α]_D_^25^ −152 (c 0.03, MeOH) ^1^H NMR (400 MHz, CD_3_OD) δ 7.30 (d, *J* = 8.4 Hz, 2H), 6.80 (d, *J* = 8.4 Hz, 2H), 6.02 (s, 2H), 5.34 (dd, *J* = 12.9, 3.0 Hz, 1H), 3.79 (s, 3H), 3.12 (dd, *J* = 17.2, 13.1 Hz, 1H), 2.69 (dd, *J* = 17.2, 3.0 Hz, 1H); ^13^C NMR (100 MHz, CD_3_OD) δ 196.8, 168.1, 163.8, 163.3, 157.7, 129.5, 127.7, 114.9, 102.6, 94.3, 93.6, 79.2, 54.9, 42.6; HR-ESI-MS positive mode, *m*/*z* 287.0917, [M+H]^+^ (calcd. C_16_H_15_O_5_^+^ 287.0914, error 1.04 ppm).

### 4.3. Laxative Activity

This activity was evaluated using a larval zebrafish model. The maintenance of adult zebrafish and the collection of eggs or larvae were performed following previously established protocols from our research group [41]. To assess laxative activity, we employed a previously reported method [18] with slight modifications. The test compound was dissolved in DMSO and then diluted into E3 embryo medium for zebrafish larvae to the indicated final concentration, with a final DMSO content of 0.1% (*v*/*v*). Zebrafish eggs were collected from the natural spawning of adult zebrafish. During both egg incubation and bioactivity testing, the E3 embryo medium was replaced every 24 h. At 5 dpf, the E3 medium was replaced with 0.01 μg/mL Nile Red staining solution, and larvae were incubated for 16 h in the dark, then rinsed three times with E3. They were randomly distributed into a 96-well plate and exposed for 6 h to either 0.1% DMSO/E3 (control) or E3 containing the test compound at various concentrations. The larvae were rinsed three times with fresh E3 medium, then anesthetized with 10 µL of 1.6% tricaine (MS-222) per well, uniformly positioned, and imaged under a green laser stereomicroscope (Nikon Corporation, Tokyo, Japan). ImageJ (version 4.5) was used for fluorescence quantification.

### 4.4. Assessment of Lipid Accumulation

Lipid accumulation was determined according to a reported procedure [42]. Zebrafish larvae (2 dpf) were divided into three groups, which were exposed to blank control, 10 µM Sakuranetin, and 20 µM Sakuranetin. Three replicates were established for each experimental group, each containing 10 zebrafish larvae. After three days of treatment, the larvae were washed three times with PBS buffer and fixed in 4% paraformaldehyde for 4 h. They were then rinsed twice with PBS buffer, dehydrated in 60% propylene glycol for 20 min, and stained with 1% Oil Red O in a dark environment for 30 min. After being stained, the larvae were washed once with 60% propylene glycol and twice with PBS buffer. Images were captured under a stereo microscope (Nikon Corporation, Tokyo, Japan). ImageJ (version 4.5) was used for fluorescence quantification.

### 4.5. Metabolomic Analysis

Zebrafish larvae (2 dpf) were randomly divided into two groups: blank control group (G0) and 10 μM and 20 μM Sakuranetin-exposed groups (G1, G2). Each group contained five replicates, with 100 zebrafish per replicate. The control group (G0) was kept under the same conditions without Sakuranetin treatment. After being exposed for 3 days, the larvae were washed three times with fresh E3 water and then frozen immediately in liquid nitrogen. The frozen samples were then lyophilized using a freeze-dryer of Biosafer-10A (Nanjing Biosafer Biotechnology Co., Ltd., Nanjing, China) to remove moisture and ensure stability during storage and processing. The lyophilized samples were mixed with 80% methanol at a solid–liquid ratio of 1:10. The mixture was then ultrasonicated using a JY92-IIDN cell disruptor (Ningbo Scientz Biotechnology Co., Ltd., Ningbo, China) at 15% power to break down the samples and facilitate efficient extraction of metabolites. This procedure was carried out in an ice bath. The homogenate was centrifuged at 12,000 r/min for 20 min at 4 °C. The resulting supernatant was subsequently subjected to metabolomic analysis using a Thermo Q Exactive Focus LC-MS system (Thermo Fisher Scientific Inc., Waltham, MA, USA). Metabolite separation was performed on a Syncronis aQ column (1.7 μm, 100 mm × 2.1 mm, Thermo Fisher Scientific, Waltham, MA, USA). Metabolites were eluted with a gradient of acetonitrile (5–100%, 0.1% acetic acid) at a flow rate of 0.3 mL/min for 20 min. MS spectra were acquired in accordance with a previously described dd-MS2 module protocol [43]. The scanning was conducted separately in positive and negative ion modes, with a scan range for full MS and MS/MS of 70–1000 *m*/*z*. The resolution for full MS was 70,000, and for MS/MS, it was 35,000. Collision energies were set at 10, 20, and 40 V.

Raw data files were processed using MS-Dial 5.5 [44]. Thresholds for MS1 and MS2 were set at 0.01 and 0.05, respectively. A sigma window value of 0.5 was used for deconvolution. Additionally, the minimum threshold for peak detection was set at a height of 2 × 10^5^ and a width of 5. An MSP file was created as a reference database by referencing the KEGG compound database [45], incorporating experimental MS spectra from HMDB (5.0) [46], and using the CFM-ID package [47] to predict spectra. The mass tolerances for MS1 and MS2 were set at 2 mDa and 5 mDa, respectively. Metabolites were identified based on matching precursor *m*/*z* and MS/MS spectra. Data from QC (quality control) samples were used for reference and normalized using LOWESS. Principal component analysis (PCA) and sparse partial least squares discriminant analysis (sPLS-DA) were performed using the mixOmics package (version 6.31) [48]. Differential metabolites were filtered using criteria of FC (fold change) >2.0 and *p* < 0.01. Finally, pathway enrichment was conducted using the FELLA package (v1.16.0) [49].

### 4.6. Transcriptomic Analysis

Zebrafish larvae (3 dpf) from the blank (G0) and 10 μM Sakuranetin-exposed (G1) groups were subjected to three independent biological replicates. Transcriptome sequencing was performed at BGI Genomics (BGI Genomics Co., Ltd., Shenzhen, China) following standard protocols. Briefly, total RNA was extracted from frozen zebrafish larvae using Trizol reagent (Thermo Fisher Scientific, Waltham, MA, USA) according to the manual instructions. The quality and quantity of RNA were assessed using a NanoDrop spectrophotometer (Thermo Fisher Scientific, Wilmington, DE, USA) and an Agilent 2100 Bioanalyzer (Agilent Technologies, Santa Clara, CA, USA). The raw sequencing data were filtered using SOAPnuke (v1.5.2) to generate clean reads in FASTQ format. These data were then aligned with the reference genome using HISAT2 (v2.0.4) and with the reference coding gene set using Bowtie2 (v2.2.5). Differential expression analysis was performed using the DESeq2 (v1.4.5) tool with criteria of Q-value ≤ 0.01 and fold change (FC) ≥ 2. The data were visualized using the R package ggplot2 (version 3.5.1).

### 4.7. RT-qPCR

The Sakuranetin (10 µM) treatments in zebrafish larvae were consistent with the protocols used for the transcriptome experiments. Three biological replicates were performed for each group. RNA extraction was performed using the AG RNAex Pro reagent. The samples were homogenized in RNAex, and then an extraction agent was added before centrifugation. Following this, the supernatant was treated with chloroform and centrifuged again. Isopropanol was introduced to precipitate the RNA, which was then washed with ethanol and reconstituted in RNase-free water. The concentration of RNA was determined using a nucleic acid protein detector by calculating the A260/A280 ratio. Reverse transcription was carried out with the EVO M-MLV RT Mix kit (Accurate Biotechnology Co., Ltd., Changsha, China), which included the removal of genomic DNA, allowing the RNA to be converted into cDNA under defined reaction conditions.

For quantitative PCR analysis, the Premix Pro Taq HS qPCR kit (Accurate Biotechnology Co., Ltd., Changsha, China) was utilized. Primers were created using the online primer design tool provided by Sangon (Sangon Biotech Co., Ltd., Shanghai, China). The reaction mixture included 2X SYBR Green Pro Taq HS Premix, cDNA, forward and reverse primers, and RNase-free water. The cycling conditions for qPCR comprised an initial denaturation step at 95 °C for 30 s, followed by 40 cycles of 95 °C for 5 s and 60 °C for 30 s, concluding with a final dissociation phase.

### 4.8. Molecular Docking

The three-dimensional structures of human proteins were obtained from the AlphaFold database (accessed on 26 May 2025) [22] and converted to pdbqt format. The structure-optimized Sakuranetin molecule was also converted to PDBQT format. Binding pockets were automatically identified using PocketVina (version 0.1.0) [23], and molecular docking was performed for Sakuranetin against each predicted pocket of every protein. Docking results for each pocket were ranked according to binding energies, and for each protein, only the best binding pose (with the lowest binding energy) was retained for further analysis. The optimal protein–ligand binding conformations were visualized using the open-source software PyMOL-open-source (version 3.2) [50]. Additionally, protein–ligand interaction analyses were performed and visualized using LigPlot+ (version 2.2) [51].

## 5. Conclusions

In zebrafish larvae, a sensitive and tractable model, bioactivity-guided isolation identified Sakuranetin as the principal laxative component of *P. persica*, with a clear dose–response between 10–25 μM. Metabolomics indicated modulation of glycine, serine, and threonine metabolism. Transcriptomics and molecular docking supported engagement of gnat1 with the downstream reduction of rho expression. These changes align with enhanced intestinal peristalsis and modulation of intestinal inflammation, providing a mechanistic basis for constipation relief. Collectively, Sakuranetin emerges as a natural candidate for patients with constipation and for maintaining intestinal health. Further studies are needed to confirm target engagement in mammalian models, characterize pharmacokinetics and safety, and establish efficacy in constipation.

## Data Availability

The data presented in this study are available on request from the corresponding author.

## Supplementary Materials

The following supporting information can be downloaded at: https://www.mdpi.com/article/10.3390/ijms26178112/s1.
