# Supplementary material for: Sakuranetin, A Laxative Component from Peach Leaves and Its Intervention in Metabolism
Source: Int J Mol Sci. 2025 Aug 22;26(17):8112. doi: 10.3390/ijms26178112 (PMC12428585; doi:10.3390/ijms26178112)
Supplement: Supplementary file 1 [file ijms-26-08112-s001.zip › ijms-3781357-supplementary.pdf]

Supplementary materials

Sakuranetin, A Laxative Component from Peach Leaves and Its Intervention in Metabolism

Haixin Jiang[1] †, Ping Wang[1] †, Yi Song[1], Chenyuan Qi[1], Xubo Zhang[1], Disheng Wang[1], Luqi Li[2] and Qiang Zhang[1,*]

[1] Shaanxi Key Laboratory of Natural Products & Chemical Biology, College of Chemistry & Pharmacy, Northwest A&F University, Yangling 712100, China.

[2] Life Science Research Core Services, Northwest A&F University, Yangling 712100, China

† These authors contributed equally.

* Correspondence: zhangq@nwsuaf.edu.cn (Q. Z.)

Contents

Figure S1. Morphological appearance of Peach (*Prunus persica*) Leaves 2

Figure S2. ^{1}H (400 MHz),^{13}C (100 MHz) NMR and HR−ESI−MS spectrum of Sakuranetin. 2

Table S1. Metabolites Identified by MS/MS in Positive Ion Mode 4

Table S2. Metabolites Identified by MS/MS in Negative Ion Mode 7

Table S3. Spearman correlation analysis of changed metabolites influenced by Sakuranetin 10

Table S4. Differentially expressed genes in the transcriptome 11

Figure S1. Morphological appearance of Peach (*Prunus persica*) Leaves

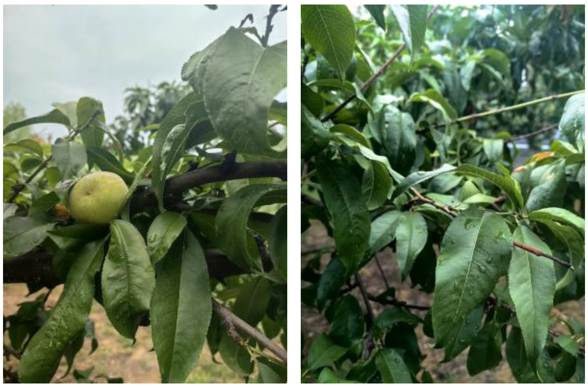

Figure S2. ^{1}H (400 MHz),^{13}C (100 MHz) NMR and HR−ESI−MS spectrum of Sakuranetin.

(1) ^{1}H−NMR

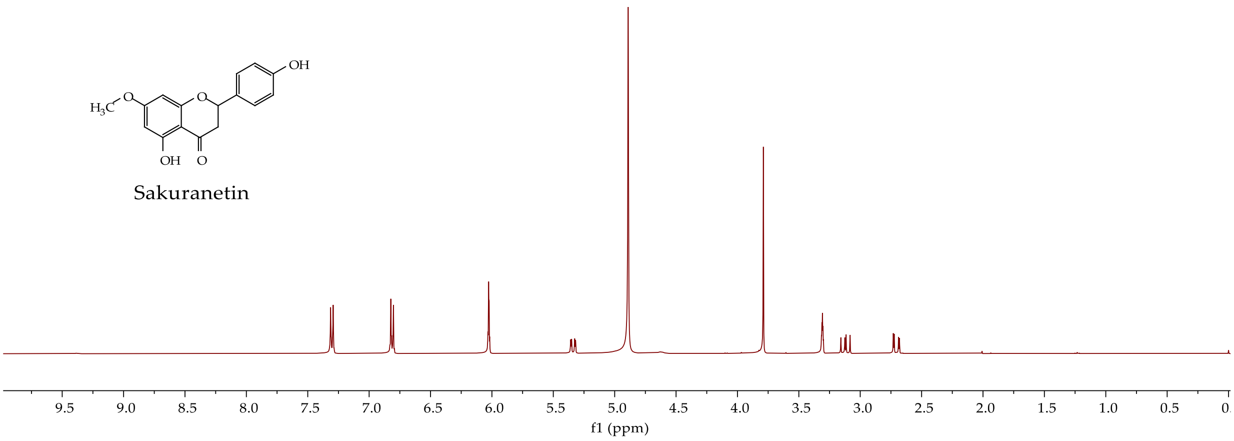

(2) ^{13}C−NMR

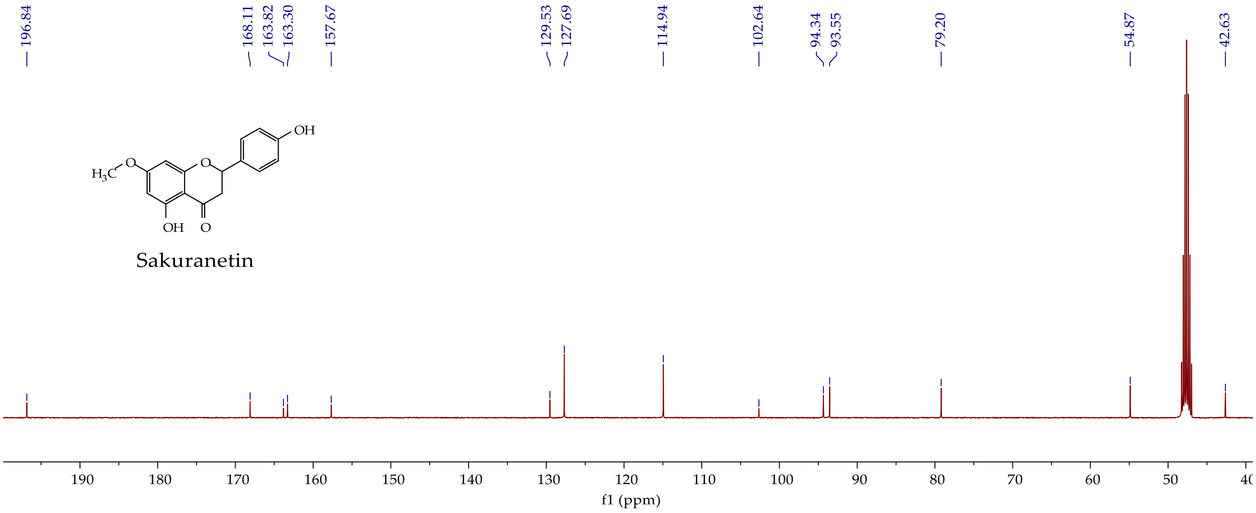

(3) HR−ESI MS

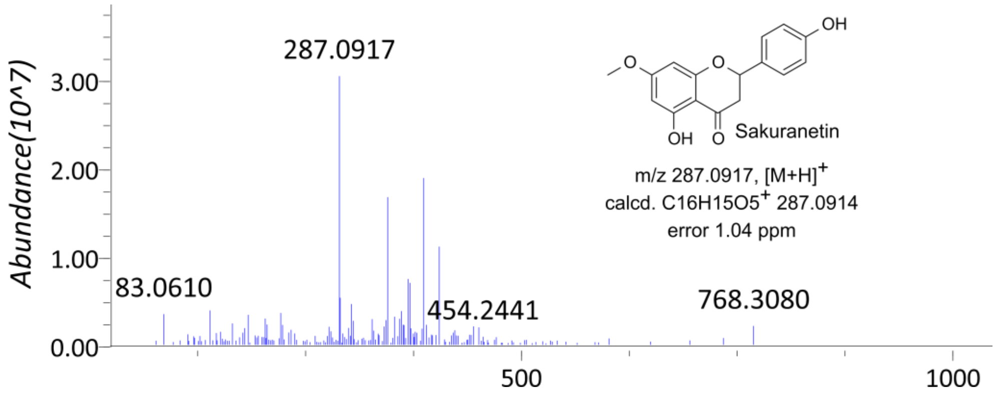
287.0917
83.0610
454.2441
768.3080
Abundance(10^7)
3.00
2.00
1.00
0.00
500
1000
OH
O
O
OH
O
Sakuranetin
m/z 287.0917, [M+H]+
calcd. C16H15O5+ 287.0914
error 1.04 ppm

Table S1. Metabolites Identified by MS/MS in Positive Ion Mode

	m/z	Formula	Adduct type	KEGG ID	name
1	72.08119	$C_5H_{11}NO_2$	$[M-CHO_2]^+$	C00183	L−valine
2	79.02154	C_2H_6OS	$[M+H]^+$	C11143	Dimethyl sulfoxide
3	83.03786	$C_5H_7NO_3$	$[M-CH_2O_2]^+$	C02237	5−Oxo−D−proline
4	97.99154	$C_5H_4N_2O_4$	$[M+Ca]^{2+}$	C00295	Orotic acid
5	98.98438	H_3O_4P	$[M+H]^+$	C00009	Ortophosphate
6	101.07115	$C_5H_{11}N_3O_2$	$[M-CH_2NO]^+$	C01035	4−Guanidinobutyric acid
7	102.03403	$C_5H_8O_5$	$[M-CH_2O_2]^+$	C02630	2−hydroxyglutaric acid
8	110.02014	$C_9H_{12}N_2O_6$	$[M-C_5H_4N_5]^+$	C00299	Uridine
9	113.03458	$C_4H_4N_2O_2$	$[M+H]^+$	C00106	Uracil
10	116.07055	$C_5H_9NO_2$	$[M+H]^+$	C00148	L−Proline
11	118.08618	$C_5H_{11}NO_2$	$[M+H]^+$	C00719	Betaine
12	120.08076	$C_8H_{11}NO$	$[M-OH]^+$	C02735	Phenylethanolamine
13	122.09639	$C_8H_{11}N$	$[M+H]^+$	C11004	2,6−Xylidine
14	123.0403	$C_{14}H_{12}O_2$	$[M-5H_2O+H]^+$	C01408	Benzoin
15	123.04394	$C_8H_9NO_3$	$[M-CH_2NO]^+$	C12323	L−4−Hydroxyphenylglycine
16	123.05522	$C_6H_6N_2O$	$[M+H]^+$	C00153	Nicotinamide
17	127.05012	$C_5H_6N_2O_2$	$[M+H]^+$	C00178	Thymine
18	130.04973	$C_5H_{10}N_2O_3$	$[M-NH_2]^+$	C00819	D−Glutamine
19	130.06499	C_9H_7N	$[M+ACN+H]^+$	C06413	Quinoline
20	130.08615	$C_6H_{14}N_2O_2$	$[M+H-NH_3]^+$	C00047	L−Lysine
21	132.07648	$C_4H_9N_3O_2$	$[M+H]^+$	C00300	Creatine
22	132.10173	$C_6H_{13}NO_2$	$[M+H]^+$	C21096	L−allo−Isoleucine
23	133.03149	$C_5H_{11}NO_2S$	$[M-NH_2]^+$	C00073	L−Methionine
24	136.06158	$C_5H_5N_5$	$[M+H]^+$	C00147	Adenine
25	136.07545	$C_9H_{11}NO_3$	$[M-CHO_2]^+$	C00082	L−Tyrosine
26	136.07545	C_8H_9NO	$[M+H]^+$	C15561	N−Benzylformamide
27	137.04555	$C_5H_4N_4O$	$[M+H]^+$	C00262	Hypoxanthine
28	139.01759	$C_{10}H_8O_4$	$[M-3H_2O+H]^+$	C18079	Isoscopoletin
29	139.01767	$C_{16}H_{12}O_5$	$[M-C_6H_{10}O_4+H]^+$	C14536	Glycitein
30	139.04993	$C_6H_6N_2O_2$	$[M+H]^+$	C00785	Urocanate
31	144.98195	$C_6H_6O_3$	$[M-H_2N+Cl]^+$	C01108	1,2,3−Trihydroxybenzene
32	147.0437	$C_9H_6O_2$	$[M+H]^+$	C05851	Coumarin
33	148.06007	$C_5H_9NO_4$	$[M+H]^+$	C00025	L−Glutamic acid
34	149.02303	$C_6H_{12}O_6$	$[M-CH_5N]^+$	C00137	inositol
35	149.02303	$C_2H_8NO_3P$	$[M+H+Na]^+$	C03557	2−Aminoethylphosphonate
36	149.0231	$C_{10}H_{11}NO_4$	$[M-C_2H_6NO]^+$	C05596	28116−23−6
37	149.02312	$C_{10}H_8O_5$	$[M-C_2H_3O_2]^+$	C20772	3−[(1−Carboxyvinyl)oxy]benzoic acid
38	152.05643	$C_5H_5N_5O$	$[M+H]^+$	C00242	Guanine
39	153.0405	$C_5H_4N_4O_2$	$[M+H]^+$	C00385	Xanthine
40	154.00476	$C_6H_{10}O_6$	$[M-C_2H_3O_2+Cl]^+$	C00198	gluconolactone
41	154.98985	$C_{13}H_8N_2O_2$	$[M-C_5H_9]^+$	C21442	phenazine−1−carboxylic acid
42	155.97374	$C_{20}H_{21}FN_2O$	$[M-C_{14}H_{23}+Na]^+$	C07572	citalopram

43	156.07642	$C_6H_9N_3O_2$	$[M+H]^+$	C00135	L−Histidine
44	156.07648	$C_{10}H_{13}N_5O_4$	$[M-C_4H_3N_2O_2]^+$	C00330	2'−deoxyguanosine
45	158.0121	$C_8H_8O_2$	$[M-HO+K]^+$	C00548	Phenyl acetate
46	158.01213	$C_7H_6O_3$	$[2M+Ca]^{2+}$	C00805	Salicylate
47	158.01216	$C_8H_8O_2$	$[M-HO+K]^+$	C07215	o−Toluate
48	162.11205	$C_7H_{15}NO_3$	$[M+H]^+$	C00318	L−Carnitine
49	164.06659	$C_6H_{12}O_6$	$[M-HO+H]^+$	C06153	inositol
50	165.05052	$C_{13}H_{16}N_2O_4$	$[M-C_6H_6N_2O+Na]^+$	C04148	Phenylacetylglutamine
51	165.0506	$C_6H_{14}O_6$	$[M-H_3N]^+$	C01697	Galactitol
52	166.08591	$C_9H_{11}NO_2$	$[M+H]^+$	C02265	D−Phenylalanine
53	169.03525	$C_5H_4N_4O_3$	$[M+H]^+$	C00366	Uric acid
54	175.11861	$C_6H_{14}N_4O_2$	$[M+H]^+$	C00062	L−Arginine
55	180.04417	$C_8H_8O_3$	$[M-HO+HCOO]^+$	C05593	3−Hydroxyphenylacetic acid
56	181.02805	$C_7H_8O_2$	$[M+H_2O+K]^+$	C17467	p−Hydroxybenzyl alcohol
57	181.02806	$C_{15}H_{10}O_3$	$[M-C_3H_5O]^+$	C01495	Flavonol
58	181.02811	$C_6H_{10}N_2O_2$	$[M+Ca-H]^+$	C06231	ectoine
59	182.0808	$C_9H_{10}O_4$	$[M+NH_4]^+$	C10447	3−(3,4−dihydroxyphenyl)propanoic acid
60	184.07307	$C_5H_{15}NO_4P$	$[M]^+$	C00588	O−Phosphocholine
61	185.02098	$C_{11}H_{12}N_2O_3$	$[M-C_2H_4NO_2+K]^+$	C00643	5−hydroxy−L−tryptophan
62	186.99501	$C_4H_6O_4$	$[M+{}_3Na]^+$	C00042	Succinate
63	188.0701	$C_{11}H_{12}N_2O_2$	$[M-NH_2]^+$	C00078	L−Tryptophan
64	190.0495	$C_4H_9NO_2S$	$[M+MeOH+Na]^+$	C05330	Homocysteine
65	192.13777	C_9H_9N	$[M+iPrOH+H]^+$	C08313	3−METHYLINDOLE
66	195.00513	$C_{10}H_{11}NO$	$[M-CHO_2+Br]^+$	C00955	Tryptophol
67	197.00035	$C_{15}H_{10}O_5$	$[M-C_3H_7NO]^+$	C06563	Genistein
68	197.00037	$C_{15}H_{10}O_5$	$[M-C_3H_7NO]^+$	C01477	Apigenin
69	197.00038	$C_{15}H_{10}O_6$	$[M-{}_5H_2O+H]^+$	C05903	Kaempferol
70	197.00046	$C_{15}H_{10}O_6$	$[M-{}_5H_2O+H]^+$	C01514	Luteolin
71	197.00046	$C_4H_6N_4O_3$	$[M+Ca-H]^+$	C01551	Allantoin
72	197.01959	$C_{10}H_{12}N_4O_6$	$[M-C_3H_7N_2O]^+$	C01762	Xanthosine
73	198.08685	$C_8H_{11}N_3O_3$	$[M+H]^+$	C02997	N−Acetylhistidine
74	199.03857	$C_{13}H_{16}N_2O_2$	$[M-C_3H_6NO+K]^+$	C01598	Melatonin
75	204.1226	$C_9H_{18}NO_4$	$[M+H]^+$	C02571	Acetyl−L−carnitine
76	205.09671	$C_{11}H_{12}N_2O_2$	$[M+H]^+$	C00525	D−Tryptophan
77	219.19493	$C_{12}H_{24}O_2$	$[M+H_2O+H]^+$	C02679	lauric acid
78	220.11739	$C_9H_{17}NO_5$	$[M+H]^+$	C00864	D−Pantothenic acid
79	222.00459	$C_{11}H_9NO_3$	$[M-H_2N+Cl]^+$	C00331	Indolepyruvate
80	223.07419	$C_7H_{14}N_2O_4S$	$[M+H]^+$	C00542	cystathionine
81	228.19522	$C_5H_{10}O$	$[2M+3H_2O+2H]^+$	C01390	Prenol
82	238.09294	$C_9H_{11}N_5O_3$	$[M+H]^+$	C06313	Biopterin
83	240.10851	$C_9H_{13}N_5O_3$	$[M+H]^+$	C02953	7,8−Dihydrobiopterin
84	252.10855	$C_{10}H_{13}N_5O_3$	$[M+H]^+$	C05198	5'−Deoxyadenosine
85	266.09509	$C_{12}H_{15}N_3O_2S$	$[M+H]^+$	C01779	Albendazole
86	268.10333	$C_{10}H_{13}N_5O_4$	$[M+H]^+$	C00212	Adenosine

87	268.99777	$C_{15}H_{12}O_6$	$[M-C_2H_4NO+K]^+$	C05631	Eriodictyol
88	269.0802	$C_{16}H_{12}O_4$	$[M+H]^+$	C00858	Formononetin
89	269.08734	$C_{10}H_{12}N_4O_5$	$[M+H]^+$	C00294	Inosine
90	273.07495	$C_{15}H_{12}O_5$	$[M+H]^+$	C06561	Naringenin chalcone
91	274.27325	$C_{16}H_{32}O_2$	$[M+H]^+$	C00249	Palmitic Acid
92	279.15836	$C_7H_{12}O_2$	$[2M+Na]^+$	C09822	Cyclohexane−1−carboxylate
93	279.15839	$C_{13}H_{20}O_3$	$[M+MeOH+Na]^+$	C01760	Vomifoliol
94	282.2782	$C_{18}H_{39}NO_3$	$[M-_2H_2O+H]^+$	C12144	phytosphingosine
95	284.09824	$C_{10}H_{13}N_5O_5$	$[M+H]^+$	C00387	Guanosine
96	285.07507	$C_{16}H_{12}O_5$	$[M+H]^+$	C01470	Acacetin
97	285.22034	$C_{36}H_{60}O_2$	$[M+_2Na]^{2+}$	C02588	Retinyl palmitate
98	285.22058	$C_{20}H_{28}O$	$[M+H]^+$	C00376	All trans retinal
99	287.09048	$C_{16}H_{14}O_5$	$[M+H]^+$	C09833	Sakuranetin
100	290.26819	$C_{16}H_{30}O_2$	$[M+H_2O+NH_4]^+$	C08362	(9Z)−Hexadecenoic acid
101	298.09604	$C_{11}H_{15}N_5O_3S$	$[M+H]^+$	C00170	5'−Methylthioadenosine
102	307.08231	$C_{15}H_{14}O_7$	$[M+H]^+$	C12136	Epigallocatechin
103	308.09024	$C_{10}H_{17}N_3O_6S$	$[M+H]^+$	C00051	Glutathione
104	318.29941	$C_{18}H_{37}NO_2$	$[M+H_2O+H]^+$	C00319	sphingosine
105	348.06934	$C_{10}H_{14}N_5O_7P$	$[M+H]^+$	C00020	AMP
106	349.03552	$C_{10}H_{12}N_5O_6P$	$[2M+Ca]^{2+}$	C00575	Cyclic AMP
107	349.05341	$C_{10}H_{13}N_4O_8P$	$[M+H]^+$	C00130	INOSINE−MONOPHOSPHATE
108	352.31973	$C_{20}H_{38}O_2$	$[M+ACN+H]^+$	C16526	cis−11−eicosenoic acid
109	355.10165	$C_{16}H_{18}O_9$	$[M+H]^+$	C00852	Chlorogenic acid
110	357.27771	$C_{24}H_{40}O_4$	$[M-_2H_2O+H]^+$	C02528	chenodeoxycholic acid
111	362.32504	$C_{18}H_{39}NO_2$	$[M+CH_3COOH+H]^+$	C00836	Sphinganine
112	362.32538	$C_{10}H_{20}O_2$	$[2M+NH4]^+$	C01571	Decanoic acid
113	364.06439	$C_{10}H_{14}N_5O_8P$	$[M+H]^+$	C00144	GUANOSINE MONOPHOSPHATE
114	385.12787	$C_{14}H_{20}N_6O_5S$	$[M+H]^+$	C00021	S−Adenosyl−L−homocysteine
115	398.23138	$C_{20}H_{32}O_5$	$[M+HCOO+H]^+$	C00584	BETA−SITOSTEROL
116	400.34106	$C_{23}H_{45}NO_4$	$[M+H]^+$	C02990	Palmitoyl−L−Carnitine
117	417.11688	$C_{21}H_{20}O_9$	$[M+H]^+$	C10524	Puerarin
118	428.03571	$C_{10}H_{15}N_5O_{10}P_2$	$[M+H]^+$	C00054	"ADENOSINE 3',5'−DIPHOSPHATE"
119	433.11203	$C_{21}H_{20}O_{10}$	$[M+H]^+$	C04608	Apigenin−7−O−glucoside
120	447.12756	$C_{22}H_{22}O_{10}$	$[M+H]^+$	C05376	Biochanin A−beta−D−glucoside
121	453.34229	$C_{29}H_{50}O$	$[M+Ca-H]^+$	C01753	beta−Sitosterol
122	489.31189	$C_{33}H_{38}N_4O_6$	$[M-H_2O_4P]^+$	C16641	irinotecan
123	563.17261	$C_{12}H_{15}N_3O_3S$	$[2M+H]^+$	C02809	Albendazole sulfoxide

Table S2. Metabolites Identified by MS/MS in Negative Ion Mode

	Average Mz	Formula	Adduct type	kid	name
1	71.01253	$C_3H_4O_2$	$[M-H]^-$	C00511	C00511
2	75.00741	$C_8H_8O_5$	$[M-C_6H_5O_2]^-$	C05580	3,4−Dihydroxymandelic acid
3	88.03902	$C_3H_7NO_2$	$[M-H]^-$	C00041	L−ALANINE
4	91.02077	$C_3H_8O_3$	$[M-H]^-$	C00116	Glycerol
5	96.95882	H_3O_4P	$[M-H]^-$	C00009	Phosphoric acid
6	104.03401	$C_3H_7NO_3$	$[M-H]^-$	C00740	D−Serine
7	114.05471	$C_5H_9NO_2$	$[M-H]^-$	C00763	D−proline
8	116.04932	$C_9H_7NO_2$	$[M-CHO_2]^-$	C19837	Indole−3−carboxylate
9	117.01807	$C_4H_6O_4$	$[M-H]^-$	C00042	Succinic acid
10	118.04968	$C_4H_9NO_3$	$[M-H]^-$	C00188	Threonine
11	121.02827	$C_7H_6O_2$	$[M-H]^-$	C00633	4−Hydroxybenzaldehyde
12	121.0283	$C_8H_8O_3$	$[M-CH_3O]^-$	C00755	4−Hydroxy−3−methoxy−benzaldehyde
13	124.00614	$C_2H_7NO_3S$	$[M-H]^-$	C00245	Taurine
14	130.04974	$C_5H_9NO_3$	$[M-H]^-$	C01073	C01073
15	131.03378	$C_5H_8O_4$	$[M-H]^-$	C00489	Glutaric acid
16	131.08138	$C_5H_{12}N_2O_2$	$[M-H]^-$	C00077	L−Ornithine
17	132.02908	$C_4H_7NO_4$	$[M-H]^-$	C00049	L−Aspartate
18	133.01299	$C_4H_6O_5$	$[M-H]^-$	C00149	(S)−Malate
19	134.0461	$C_5H_5N_5$	$[M-H]^-$	C00147	Adenine
20	135.03003	$C_5H_4N_4O$	$[M-H]^-$	C00262	Hypoxanthine
21	135.04399	$C_8H_8O_2$	$[M-H]^-$	C10700	4'−Hydroxyacetophenone
22	137.02328	$C_7H_6O_3$	$[M-H]^-$	C00156	4−Hydroxybenzoic acid
23	140.01071	$C_2H_8NO_4P$	$[M-H]^-$	C00346	C00346
24	145.04948	$C_6H_{10}O_4$	$[M-H]^-$	C06104	Adipic acid
25	146.04471	$C_5H_9NO_4$	$[M-H]^-$	C00025	Glutamic acid
26	149.00848	$C_4H_6O_6$	$[M-H]^-$	C02107	L−(+)−Tartaric acid
27	150.04099	$C_5H_5N_5O$	$[M-H]^-$	C00242	Guanine
28	150.04102	$C_{10}H_{13}N_5O_4$	$[M-C_5H_9O_3]^-$	C00330	2'−deoxyguanosine
29	151.03896	$C_8H_8O_3$	$[M-C_6H_{10}O_5-H]^-$	C12305	Methyl salicylate
30	151.03897	$C_9H_{10}O_4$	$[M-CH_3O]^-$	C05582	Homovanillic acid
31	152.08177	$C_7H_{11}N_3O$	$[M-H]^-$	C05135	N−Acetylhistamine
32	154.06107	$C_6H_9N_3O_2$	$[M-H]^-$	C00135	L−HISTIDINE
33	159.0652	$C_7H_{12}O_4$	$[M-H]^-$	C02656	Pimelic acid
34	163.039	$C_9H_8O_3$	$[M-H]^-$	C00166	Phenylpyruvic acid
35	164.07062	$C_{11}H_{13}NO_3$	$[M-C_2H_3O]^-$	C03519	N−Acetyl−L−phenylalanine
36	164.07071	$C_9H_{11}NO_2$	$[M-H]^-$	C00079	Phenylalanine
37	165.05476	$C_9H_{10}O_3$	$[M-H]^-$	C05607	D(+)−Phenyllactic acid
38	167.01996	$C_5H_4N_4O_3$	$[M-H]^-$	C00366	URATE
39	171.00536	$C_3H_9O_6P$	$[M-H]^-$	C00093	C00093
40	171.06525	$C_5H_4O_3$	$[M+iPrOH-H]^-$	C01546	2−Furoate
41	174.0397	$C_{11}H_{16}N_2O_8$	$[M-C_5H_8NO_3]^-$	C12270	N−Acetylaspartylglutamate
42	175.02377	$C_6H_8O_6$	$[M-H]^-$	C00072	L−Ascorbic acid

43	176.93491	$H_4O_7P_2$	$[M-H]^-$	C00013	LTS0095310
44	178.03606	$C_6H_5N_5O_2$	$[M-H]^-$	C03975	Isoxanthopterin
45	180.06561	$C_9H_{10}INO_3$	$[M-I]^-$	C02515	3−Iodo−L−tyrosine
46	181.07076	$C_6H_{14}O_6$	$[M-H]^-$	C00794	D−Sorbitol
47	183.01508	$C_6H_4N_2O_5$	$[M-H]^-$	C02496	2,4−Dinitrophenol
48	188.05539	$C_7H_{11}NO_5$	$[M-H]^-$	C00624	N−Acetyl−L−glutamate
49	190.05011	$C_{10}H_9NO_3$	$[M-H]^-$	C05635	C05635
50	191.01874	$C_6H_8O_7$	$[M-H]^-$	C00158	Citrate
51	191.01874	$C_4H_4O_5$	$[M+CH_3COO]^-$	C00036	Oxaloacetate
52	196.07181	$C_8H_{11}N_3O_3$	$[M-H]^-$	C02997	N−Acetylhistidine
53	201.0248	$C_{12}H_{22}O_{11}$	$[M-C_8H_{13}O_2]^-$	C00208	Maltose
54	201.03717	$C_6H_{12}O_6$	$[M+Na-2H]^-$	C00137	inositol
55	203.08179	$C_{11}H_{12}N_2O_2$	$[M-H]^-$	C00525	D−(+)−Tryptophan
56	209.04482	$C_8H_6O_3$	$[M+CH_3COO]^-$	C02137	BENZOYLFORMIC ACID
57	217.00244	$C_8H_8N_2O_3$	$[M+K-2H]^-$	C05380	Nicotinuric acid
58	217.00246	$C_6H_7NO_3S$	$[M+HCOO-H]^-$	C06333	2−Aminobenzenesulfonate
59	218.10281	$C_9H_{17}NO_5$	$[M-H]^-$	C00864	Pantothenate
60	219.97701	$C_3H_8NO_6P$	$[M+Cl]^-$	C01005	O−Phospho−L−serine
61	223.0278	$C_5H_6O_5$	$[M+DMSO-H]^-$	C00026	2−Oxoglutaric acid
62	223.13336	$C_5H_{11}NO_2$	$[M+ACN+ACN]^-$	C00183	L−valine
63	223.13338	$C_{15}H_{22}O_5$	$[M-C_2H_3O_2]^-$	C15971	dihydrophaseic acid
64	223.13341	$C_{21}H_{32}O_5$	$[M-C_8H_{13}O_2]^-$	C05470	TETRAHYDROCORTISONE
65	229.01131	$C_5H_{11}O_8P$	$[M-H]^-$	C01112	D−Arabinose−5−phosphate
66	239.0592	$C_6H_{10}O_5$	$[M+DMSO-H]^-$	C03761	3−Hydroxy−3−methylglutarate
67	243.0618	$C_9H_{12}N_2O_6$	$[M-H]^-$	C00299	Uridine
68	251.0782	$C_{10}H_{12}N_4O_4$	$[M-H]^-$	C05512	Deoxyinosine
69	253.217	$C_{16}H_{30}O_2$	$[M-H]^-$	C08362	palmitoleic acid
70	255.23265	$C_{18}H_{30}O_2$	$[M-Na]^-$	C06427	(9Z,12Z,15Z)−Octadecatrienoic acid
71	259.02209	$C_6H_{13}O_9P$	$[M-H]^-$	C00636	alpha−D−(+)−mannose−1−phosphate
72	267.07339	$C_{10}H_{12}N_4O_5$	$[M-H]^-$	C00294	Inosine
73	271.06097	$C_{15}H_{12}O_5$	$[M-H]^-$	C00509	Naringenin
74	277.12259	$C_{11}H_{22}N_2O_4S$	$[M-H]^-$	C00831	pantetheine
75	277.14435	$C_{16}H_{22}O_4$	$[M-H]^-$	C03343	Mono(2−ethylhexyl) phthalate (MEHP)
76	277.18066	$C_{20}H_{32}O_5$	$[M-C_3H_7O_2]^-$	C00584	BETA−SITOSTEROL
77	282.08432	$C_{10}H_{13}N_5O_5$	$[M-H]^-$	C00387	Guanosine
78	283.0683	$C_{10}H_{12}N_4O_6$	$[M-H]^-$	C01762	Xanthosine
79	283.26407	$C_{18}H_{36}O_2$	$[M-H]^-$	C01530	Stearic acid
80	285.08539	$C_{11}H_{12}N_2O_2$	$[M+CH_3COONa-H]^-$	C00078	L−Tryptophan
81	288.12003	$C_{11}H_{19}N_3O_6$	$[M-H]^-$	C21016	OPHTHALMATE
82	296.08209	$C_{11}H_{15}N_5O_3S$	$[M-H]^-$	C00170	5'−Deoxy−5'−Methylthioadenosine
83	297.24338	$C_{18}H_{34}O_3$	$[M-H]^-$	C19418	FA 18:1+1O
84	301.16589	$C_7H_{12}O_2$	$[2M+HCOO]^-$	C09822	Cyclohexane−1−carboxylate
85	301.21713	$C_{20}H_{30}O_2$	$[M-H]^-$	C06428	Eicosapentaenoic Acid
86	303.23282	$C_{20}H_{32}O_2$	$[M-H]^-$	C00219	Arachidonic acid

87	305.02295	$C_9H_{14}N_4O_3$	[M+Br]⁻	C00386	Carnosine
88	306.07632	$C_{10}H_{17}N_3O_6S$	[M−H]⁻	C00051	L−Glutathione (reduced)
89	311.29544	$C_{20}H_{40}O_2$	[M−H]⁻	C06425	arachidic acid
90	322.04431	$C_9H_{14}N_3O_8P$	[M−H]⁻	C00055	CMP
91	323.0282	$C_9H_{13}N_2O_9P$	[M−H]⁻	C00105	UMP
92	337.20532	$C_{21}H_{28}O_5$	[M−Na]⁻	C00762	Cortisone
93	339.3266	$C_{22}H_{44}O_2$	[M−H]⁻	C08281	Behenic acid
94	346.05539	$C_{10}H_{15}N_5O_{10}P_2$	[M−H2O3P]⁻	C00008	ADP
95	346.0556	$C_{10}H_{15}N_5O_{11}P_2$	[M−H2O4P]⁻	C00035	GDP
96	357.13434	$C_{20}H_{22}O_6$	[M−H]⁻	C10682	Matairesinol
97	362.05054	$C_{10}H_{14}N_5O_8P$	[M−H]⁻	C00144	GUANOSINE MONOPHOSPHATE
98	369.02145	$C_{10}H_{13}N_4O_8P$	[M+Na−2H]⁻	C00130	IMP
99	375.13083	$C_{17}H_{20}N_4O_6$	[M−H]⁻	C00255	RIBOFLAVIN
100	383.11429	$C_{14}H_{20}N_6O_5S$	[M−H]⁻	C00021	S−(5'−ADENOSYL)−L−HOMOCYSTEINE
101	383.12231	$C_9H_{11}NO_3$	[2M−2H+Na]⁻	C00082	L−Tyrosine
102	402.99463	$C_9H_{14}N_2O_{12}P_2$	[M−H]⁻	C00015	URIDINE 5'−DIPHOSPHATE
103	407.28027	$C_{24}H_{40}O_5$	[M−H]⁻	C00695	Cholic acid
104	415.10355	$C_{21}H_{20}O_9$	[M−H]⁻	C10524	Puerarin
105	433.11386	$C_{21}H_{22}O_{10}$	[M−H]⁻	C09099	naringenin−7−O−glucoside
106	565.04779	$C_{15}H_{24}N_2O_{17}P_2$	[M−H]⁻	C00029	UDPG
107	579.02747	$C_{15}H_{22}N_2O_{18}P_2$	[M−H]⁻	C00167	Uridine diphosphate glucuronic acid
108	606.07471	$C_{17}H_{27}N_3O_{17}P_2$	[M−H]⁻	C00043	Uridine 5'−diphospho−N−acetylglucosamine
109	611.14465	$C_{20}H_{32}N_6O_{12}S_2$	[M−H]⁻	C00127	L−Glutathione oxidized
110	662.10266	$C_{21}H_{28}N_7O_{14}P_2$	[M−H]⁻	C00003	beta−Nicotinamide adenine dinucleotide hydrate

Table S3. Spearman correlation analysis of changed metabolites influenced by Sakuranetin

	KEGG ID	Name	Separman correlation	−log(Q*)	monotonicity	LC−MS ion mode
1	C00584	Prostaglandin E2	0.89	3.70	up	positive
2	C00858	Formononetin	0.95	5.33	up	positive
3	C01470	Acacetin	0.86	3.12	up	positive
4	C04608	Apigetrin	0.85	3.04	up	positive
5	C05376	Sissotrin	0.95	5.33	up	positive
6	C06313	Biopterin	0.91	4.12	up	positive
7	C06561	Naringenin chalcone	0.94	5.33	up	positive
8	C09833	Sakuranetin	0.93	4.64	up	positive
9	C00009	Orthophosphate	0.94	5.68	up	negative
10	C00013	Diphosphate	0.94	5.68	up	negative
11	C00029	UDP−glucose	0.94	5.68	up	negative
12	C00035	GDP	0.83	3.12	up	negative
13	C00041	L−Alanine	0.91	4.47	up	negative
14	C00043	UDP−N−acetylglucosamine	0.89	4.08	up	negative
15	C00072	Ascorbate	0.89	4.08	up	negative
16	C00078	L−Tryptophan	−0.89	4.08	dn	negative
17	C00116	Glycerol	0.83	3.12	up	negative
18	C00158	Citrate	0.85	3.33	up	negative
19	C00188	L−Threonine	0.83	3.12	up	negative
20	C00386	Carnosine	0.94	5.68	up	negative
21	C00387	Guanosine	−0.81	2.89	dn	negative
22	C00509	Naringenin	0.95	5.68	up	negative
23	C00740	D−Serine	0.91	4.47	up	negative
24	C01530	Octadecanoic acid	0.93	4.93	up	negative
25	C03519	N−Acetyl−L−phenylalanine	0.81	2.89	up	negative
26	C05380	Nicotinurate	−0.83	3.12	dn	negative
27	C09099	Prunin	0.95	5.68	up	negative
28	C21016	Ophthalmate	0.91	4.47	up	negative

*: adjusted p value by False Discovery Rate (FDR) method.

Table S4. Differentially expressed genes in the transcriptome

Gene ID	Gene Symbol	log_2 (G1 / G0)	−log(Q*)
140634	cyp1a	3.04	82.18
171481	fabp10a	−2.14	3.23
335654	camk1ga	−2.07	8.62
402880	cox4i1l	2.20	6.13
406602	egln3	2.15	18.69
493589	pcdh2ab1	−5.59	2.06
553474	crybgx	−3.67	2.84
554107	ela3l	−3.99	4.56
561790	hbae5	2.04	4.14
571146	fam46ab	−2.73	3.22
574001	c6ast4	−2.54	2.38
